# Neutrophil extracellular traps and cannabinoids: potential in cancer metastasis

**DOI:** 10.3389/fonc.2025.1595913

**Published:** 2025-06-17

**Authors:** Izabela Krauze, Beata Greb-Markiewicz, Anna Kłopot, Kamila Maciejewska, Michał Bryk, Małgorzata Krzystek-Korpacka

**Affiliations:** ^1^ Department of Biochemistry and Immunochemistry, Wroclaw Medical University, Wroclaw, Poland; ^2^ Department of Biochemistry, Molecular Biology and Biotechnology, Wroclaw University of Science and Technology, Wroclaw, Poland

**Keywords:** phytocannabinoids, endocannabinoid system, neutrophils, NEtosis, cancer metastasis

## Abstract

Cancer is the second leading cause of global mortality after cardiovascular diseases, with breast, lung, colon, and prostate cancers being the most common. WHO projects around 30 million new cancer cases worldwide by 2045, with breast cancer being the most common in women and lung cancer in men. Metastasis is responsible for nearly 90% of cancer-related deaths. Breast and lung cancers tend to metastasize to the bones, lymph nodes, lungs, liver, and brain. Lungs remains one of the most common organs to which various forms of cancer metastasize. An important factor in metastasis is NETosis – it can initially help to eliminate cancer cells, but it can also promote metastasis. Phytocannabinoids, compounds derived from *Cannabis sativa*, and the endocannabinoid system (ECS) offer promising therapeutic potential to inhibit NETosis and consequently cancer development and metastasis. Although the precise effects of phytocannabinoids on neutrophil functions and NETosis are not fully understood and require further research in the context of cancer, preliminary studies suggest their potential to inhibit NET release in various disease models. This review consolidates current knowledge and provides new insights into how phytocannabinoids and the ECS may serve as effective therapeutic tools to limit cancer metastasis.

## 1 Introduction

Right after cardiovascular diseases, cancers are the second leading cause of death worldwide. The most common cancers include breast, lung, colorectal, and prostate cancer. The World Health Organization (WHO) predicts that there will be approximately 30 million new cases of cancer worldwide by 2045 (WHO, 2024). According to the World Cancer Research Fund International, in 2022 breast and lung cancers comprised 12.5% and 12.2% of newly diagnosed cases, respectively. Lung cancer was the most common in men (15.4% of new cases), while breast cancer dominated among women (25.8%). According to the GLOBOCAN, projections for 2040 estimate over 3 million new breast cancer cases and approximately 1 million deaths (1). Lung cancer followed closely, with nearly 2.5 million new cases in 2022 (2). Metastatic progression accounts for about 90% of cancer-related mortality (3, 4) with breast cancer often metastasize to bones, lymph nodes, lungs, liver, or brain (5, 6).

A critical factor facilitating metastasis is NETosis, the suicidal death of neutrophils. This process, a type of lytic cell death, results in the destruction of neutrophils and the release of neutrophil extracellular traps (NETs), which are rich in DNA and proteolytic enzymes. While components of the NET network may initially contribute to the elimination of cancer cells, chronic inflammation and excessive neutrophil activation in tumor microenvironment can lead to detrimental effects. NETs can facilitate the degradation of the extracellular matrix, thereby supporting the extravasation and transport of circulating tumor cells (CTCs). In addition, NETs can trap and anchor these cells at distant sites, thereby promoting metastasis (7). The mechanism of NET release may also enhance the epithelial-mesenchymal transition (EMT), a fundamental process in metastasis formation, and may contribute to the reactivation of dormant tumor cells (8–10).

Phytocannabinoids, together with the endocannabinoid system (ECS), represent a highly promising therapeutic avenue for attenuating neutrophil effector functions, particularly the process of NETosis. We believe that these compounds have significant potential as agents capable of effectively inhibiting metastatic progression. Phytocannabinoids, derived primarily from the *Cannabis sativa* plant, are a group of organic compounds that interact with the endocannabinoid system (ECS) in the human body. The ECS, which includes CB1R and CB2R receptors, their agonists and antagonists, and enzymes responsible for the synthesis and degradation of ligands, is closely related to the action of phytocannabinoids (11, 12). The effects of phytocannabinoids and the ECS on neutrophil effector functions, particularly NETosis, are not yet fully understood. However, there are compelling, albeit limited, data suggesting that these compounds can inhibit the release of neutrophil extracellular traps in various disease models (13–16). This inhibitory effect has not been thoroughly investigated in the context of cancer and its microenvironment.

**Figure 1:**
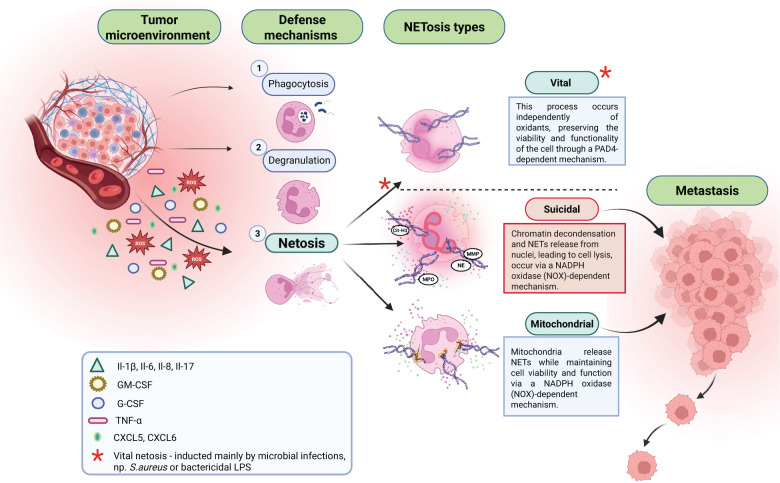
In response to agents released from the tumor microenvironment, neutrophils can present different defense mechanisms: 1. Phagocytosis; 2. Degranulation; 3. NETosis. There are three types of NETosis: vital, suicidal, and mitochondrial. Suicidal NETosis is a crucial factor supporting cancer metastasis process.

We undertook a comprehensive literature review to present the current state of knowledge, providing new insights into this topic, and illustrating how phytocannabinoids and the endocannabinoid system (ECS) could serve as exceptional therapeutic tools to limit cancer metastasis (**Figure 1**).

## 2 Tumorogenesis

The highly dynamic and protracted process of carcinogenesis involves three critical stages: initiation, promotion, and progression. During initiation, mutations arise and accumulate in genes that regulate the cell cycle, including proto-oncogenes (which are activated into oncogenes), mutator genes (responsible for maintaining genomic integrity and repairing damaged DNA), and tumor suppressor genes (which encode proteins that inhibit the cell cycle at mitosis, induce apoptosis, or mediate repair processes) (17, 18). In addition to genetic alterations, there is an accumulation of epigenetic modifications - changes in gene expression that are not associated with changes in DNA sequence, such as DNA methylation, histone modifications, and non-coding RNA (ncRNA) modifications (19, 20). The initiation process results in the formation of cancer stem cells, which exhibit unlimited proliferative potential and resistance to apoptosis-inducing factors. The subsequent phase, known as promotion, involves the clonal proliferation of these cancer stem cells, which are characterized by their ability to form colonies. In the final stage, progression, the intensive accumulation of further mutations endows cancer cells with a malignant phenotype, characterized by migratory ability and local invasion, thereby facilitating the dissemination of cancer cells and initiating metastasis (secondary cancerous lesions) (21). Research indicates that metastatic progression is responsible for most deaths caused by breast cancer, with metastatic processes accounting for nearly 90% of cancer-related mortality (3, 4, 22). Metastasis involves a cascade of sequential events. First, cancer cells detach from the stroma of the primary tumor, followed by local invasion of the surrounding tissues. Next, the cancer cells intravasate into the circulatory or lymphatic system. If the cells survive these conditions, they extravasate and colonize distant target sites (23).

## 3 Metastasis formation

The initial stage in the formation of metastases is the invasion of cancer cells into adjacent tissues and the circulatory system. This process is closely linked to the chemotactic migration of cancer cells and their morphological heterogeneity. In the context of cancer cell invasion, two different types of migration are recognized: collective and individual migration. These migration modes are intricately linked to the morphological characteristics of migrating cancer cells and the molecular genetic parameters that govern cell-cell junctions, the actin cytoskeleton, adhesion to the extracellular matrix, and protease activity (24). Epithelial cells, the origin of malignant tumors (cancers), typically exhibit collective movement characterized by tightly interconnected groups of cells that interact with the extracellular matrix. This collective migration involves an asymmetric system in which leading cells exhibit significant motor activity, while trailing cells remain relatively passive (front-back polarization). Research shows that circulating tumor cells (CTCs) more often appear as single cells than in clusters; however, clustered CTCs have a 23- to 50-fold higher metastatic potential (25). While CTCs are passively transported by the bloodstream, their collective behavior in clusters is likely more relevant to tissue migration and invasion. endot (25). Individual migration involves the movement of single cells, characterized by two distinct types of movement: mesenchymal (proteolytic) and amoeboid (non-proteolytic). Mesenchymal movement is observed in connective-tissue-derived cancer cells and in highly differentiated cells, such as those undergoing EMT transition, wherein epithelial cells acquire a mesenchymal phenotype. These cells lose their apical-basal polarity and adopt an elongated, spindle-like shape. The mesenchymal migration model includes five key steps: 1) polarization of cells into a mesenchymal phenotype and protrusions (formation of protrusions such as lamellipodia, filopodia); 2) formation of adhesive plaques at points of contact with the extracellular matrix (ECM); 3) proteolysis of ECM mediated by proteolytic enzymes; 4) reorganization of the actin cytoskeleton polarization, contraction of actin-myosin fibers which facilitates movement; and 5) trailing edge displacement toward newly formed matrix defects (26).

Cellular mobility is stimulated by various environmental factors that promote migration through sustained pro-migratory signaling via phosphatidylinositol 3-kinase (PI3K), ras-related C3 botulinum toxin substrate (Rac) or Ras homolog (Rho) pathways. In addition, migration is facilitated by factors such as autocrine motility factor (AMF), stromal-derived factor 1 (SDF1), growth factors (EGF, IGF-1), lysophosphatidic acid (LPA), and degradative enzymes such as matrix metalloproteinases (MMPs) (27). The most primitive but highly effective mode of single-cell movement is amoeboid migration. This mode is characterized by minimal or absent interaction between cells and the extracellular matrix (ECM), complete loss of cell polarity, and absence of proteolysis and expression of ECM-degrading proteolytic enzymes. Notably, cancer cells that adopt amoeboid migration exhibit the highest velocity of movement (28). In contrast to mesenchymal cells, amoeboid-moving cells exhibit a round or elliptical shape and utilize the Rho Associated Coiled-Coil Containing Protein Kinase (RHO/ROCK) signaling pathway to achieve substantial deformability, facilitating their penetration through ECM fibers (29). During cancer progression, cells can undergo phenotypic and morphological plasticity to enhance their invasive and metastatic potential, resulting in transitions between different migration modes. These transitions include mesenchymal-amoeboid, amoeboid-mesenchymal, and collective-amoeboid transitions. Of particular importance in promoting invasive potential is the transition from individual to collective migration, which is closely associated with the EMT process. During EMT, cells acquire a mesenchymal phenotype, allowing them to form cohesive cell clusters. Subsequently, these cells can revert to an epithelial phenotype through the mesenchymal to epithelial transition (MET) mechanism (30). This phenotypic flexibility allows cancer cells to adapt their migration strategies in response to environmental cues, thereby facilitating invasion and metastasis. Challenges such as undetectable micrometastases and inadequate therapeutic responses contribute significantly to the high mortality associated with metastasis initiation. The literature highlights the critical role of the tumor microenvironment, a complex milieu comprising various cellular and non-cellular components, in regulating cancer development and function. Interactions between components of the tumor microenvironment and cancer cells facilitate the acquisition of an invasive phenotype, thereby promoting the initiation of the metastatic cascade (31).

## 4 Tumor microenvironment

The tumor microenvironment (TME) functions as a specialized niche that facilitates tumorogenesis initiation and progression. It consist of various cellular components and the extracellular matrix (ECM), which includes fibrous proteins such as collagen and elastin, adhesion proteins such as fibronectin and laminin, polysaccharides such as hyaluronic acid, and glycosaminoglycans (GAGs). Among the cellular components of the TME, connective tissue elements such as fibroblasts play a critical role. Fibroblasts contribute to tumor biology by forming a barrier between the tumor and adjacent tissues and by supporting the invasive nature of cancer cells through mechanisms that include influencing angiogenesis—the process of new blood vessel formation that is critical for tumor growth and metastasis (32, 33).

In addition, the cellular components of the TME include endothelial cells, which are involved in the formation of blood and lymphatic vessels, and pericytes, which are integral to the structure of capillaries. Both endothelial cells and pericytes, situated within the tumor microenvironment, facilitate the dissemination of tumor cells through their involvement in angiogenesis, for tumor growth and metastasis (34, 35). The final component of the tumor microenvironment (TME) consists of immune system cells, including macrophages, T and B lymphocytes, neutrophils, dendritic cells, myeloid-derived suppressor cells (MDSCs), and natural killer (NK) cells. In the early stages of carcinogenesis, these immune cells exhibit anticancer activity through a variety of mechanisms. These include the cytotoxicity of NK cells, NKT cells, cytotoxic T lymphocytes, and neutrophils, as well as macrophage-mediated cytotoxicity. Additionally, they are involved in antibody-dependent cellular cytotoxicity, activation of cytokines such as interferons, interleukins, chemokines, and members of the TNF superfamily, and complement-dependent antibody cytotoxicity (36–38).

As cancer progresses, the immune system gradually loses its ability to regulate the oncogenic process. The anticancer properties of immune cells diminish, resulting in a state of immunosuppression that promotes tumor growth and metastasis. When considering the interactions of immune system cells with TME, considerable attention has traditionally been focused on macrophages and T lymphocytes (39). Recently, however, substantial evidence has emerged highlighting the pivotal role of neutrophils in cancer development, particularly in metastasis formation. Once primarily recognized for their role in acute inflammatory and infectious responses, neutrophils are now understood to play a pivotal role in oncogenesis and metastatic processes (40, 41).

## 5 Neutrophils

Neutrophils, also known as polymorphonuclear leukocytes (PMNs) comprise 50% to 70% of circulating leukocytes in peripheral blood and play a pivotal role in the innate immune response (42). Their primary function is to defend the host against pathogenic microorganisms, but they also play an important role in modulating oncogenesis (43).

Neutrophils present several defense mechanisms, including phagocytosis, intracellular degradation, degranulation, and the formation of NETs through the process of NETosis. In addition, in response to pathogenic stimuli, neutrophils secrete cytokines and various inflammatory mediators, and modulate the activity of adjacent cells. Neutrophils are a critical population within the immune system, essential for modulating inflammatory responses and serving as the first line of defense against inflammation (44). It has been reported that the neutrophil infiltration into the tumor microenvironment, characterized by chronic inflammation, is particularly intense and is mediated by various chemotactic factors. Neutrophils represent a heterogeneous population of immune cells. In the early stages of cancer, neutrophils infiltrating the tumor microenvironment often exhibit antitumor properties (N1 phenotype), engaging in direct or indirect cytotoxicity, such as the release of reactive oxygen species or neutrophil elastase, which selectively target and kill cancer cells. However, under the influence of factors secreted by cancer cells, neutrophils can switch to a pro-tumor phenotype (N2), promoting immunosuppression, inhibiting lymphoid immunity, and supporting tumor growth, angiogenesis, and metastasis, in part by remodeling of the extracellular matrix (45). Neutrophils can directly promote primary tumor progression and metastasis (44). An important effector mechanism is the production of NETs.

## 6 NETs and NETosis

Since the discovery in 1996 that NETs are produced by neutrophils, they have been intensively studied. Currently, we know that NETs are produced not only by neutrophils but also by eosinophils, mast cells and monocytes (46). NETs are DNA networks decorated with neutrophils-derived proteins: histones, granule-derived proteases such as myeloperoxidase (MPO), neutrophil elastase (NE), and cytosolic proteins: cytoplasmic calprotectin complex (S100A8/A9), lactotransferrin, azurocidin, tissue factor (TF), fibrinogen and several antimicrobial peptides (47–50). DNA present in NETs can originate from the nucleus (genomic DNA) and/or mitochondria (mitochondrial DNA) (51). Strong anti-inflammatory properties are possessed not only by proteins from granules but also by nucleic acids and histones (52).

The research showed that neutrophils originating from different organisms, e.g. mouse, fish extend their NETs in response to microorganisms. This may suggest that NETs formation by neutrophils is part of a primitive defense mechanism developed during evolution to protect organisms from infection. In addition to being involved in defense mechanisms against microorganisms in humans, NETs are also produced during non-infectious inflammatory diseases such as cancer (53), thrombosis, especially deep vein thrombosis, cystic fibrosis, or diabetes mellitus (54–56). In the tumor microenvironment, NETs production is mainly stimulated by granulocyte colony-stimulating factor (G-CSF), interleukin 8 (IL-8) and high - mobility group box 1 protein (HMGB1). It has been shown that the IL-8 is elevated in the serum of women with breast cancer (57) and that the amount of G-CSF is increased in the serum of lung cancer patients and in tissue samples from gastric cancer patients (58). IL-8 can be produced not only by tumor cells but also by endothelial cells activated by oxidative stress or inflammation as well as by stromal fibroblasts and myeloid cells present in the tumor (59). G-CSF produced by tumor cells has been shown in a mouse model to influence the presence of immune cells in the tumor, bone marrow, spleen and blood by increasing the number of neutrophils and myeloid-derived suppressor cells increases and decreasing the number of dendritic cells (60). In general, elevated levels of IL-8 and G-CSF lead to an increased number of neutrophils in the blood, their chemotaxis to the reactive oxygen species (ROS) - rich tumor microenvironment, their activation and NET production. It is well accepted that IL-8 stimulated production of NETs leads to neutrophil death (61), a process described in the literature as suicidal NETosis (7). In contrast to suicidal NETosis in vital NETosis, production of NETs by neutrophils does not lead to their death. NET production stimulated by HGMB1 may or may not lead to neutrophil death. In the case of G-CSF, it has not been determined whether NET production leads to neutrophil death (61).

IL-8 stimulated NETs generation requires an increase in intracellular calcium concentration. This process has been shown to be inhibited when either intracellular or extracellular calcium is chelated (62). It is unknown whether GCF stimulation leading to NET formation requires changes in intracellular calcium levels. It is known that an increase in intracellular calcium levels is associated with ROS generation and activation of peptidyl arginine deiminase 4 (PAD4), an enzyme responsible for citrullination of histones. NADPH oxidase and mitochondria have been shown to be a source of ROS. Fu and colleagues have shown that IL-8 stimulation of neutrophils results in activation of NADPH oxidase (63), but the role of mitochondria in this process has not been demonstrated. G-CSF has not been shown to increase NADPH oxidase activity (64), nor has it been investigated whether G-CSF stimulates mitochondrial ROS generation. It is well known that ROS generation in neutrophils causes granule rupture and release of myeloperoxidase (MPO) and neutrophil elastase (NE). NE degrades the actin cytoskeleton and translocates to the nucleus where it participates in chromatin decondensation (65). NE is not the only enzyme involved in chromatin decondensation, a hallmark of NETosis. Another enzyme involved in this process is PAD4, which is responsible for the conversion of arginine residues of histones H3, H4 and H1 to citrulline (66). This post-translational modification results in a charge change from positive to neutral, leading to a decrease in histone-DNA interaction and facilitating chromatin decondensation. Modification of lamin organization and swelling of chromatin are thought to precede a rupture of the nuclear envelope and release of nuclear contents into the cytosol in suicidal NETosis (67). The final step in this process is a rupture of the plasma membrane at multiple sites and release of NETs (68). Gasdermin D, a protein activated by NE that forms pores in the plasma membrane has been implicated in this process (69). In the case of vital NETosis, chromatin is thought to be released encapsulated in microvesicles (70). The following is a brief description of the cellular mechanisms leading to NET production and a review of the role of NETs in cancer.

### 6.1 Role of NETs in cancer

Numerous studies have shown that neutrophil extracellular traps (NETs) are present in tumor samples and may be linked to cancer development. For instance, van der Windt identified NET aggregates in pancreatic cancer tissues, while Yang et al. found NETs to be rare in primary breast tumors but abundant in liver metastases (71, 72). Elevated NETs have also been detected in the sera of patients with advanced esophageal, gastric, and lung cancers (73). Markers of NETosis, such as citrullinated histone H3 and high levels of MPO, are associated with advanced cancer stages. Concerningly, some studies suggest NETs may play a role in cancer development (8, 74–77). For example, van der Windt et al. demonstrated in a mouse model that NET formation in the liver precedes macrophage infiltration, increases in inflammatory cytokines, and tumor development (71). Inhibiting NETs with DNAse I reduced macrophage infiltration and tumor growth, suggesting that NETs may contribute to the initiation of primary liver cancer.

Tumor growth is often linked to hypoxic conditions, which strongly support NET formation. Li and colleagues tested the hypothesis that hypoxia-induced NETs accelerate gastric cancer growth (78). They found that conditioned media from hypoxia-exposed gastric cancer cells stimulated neutrophil migration and NET formation, identifying HMGB1 as a key factor and the TLR4/p38 MAPK pathway as the mechanism involved. In an *in vivo* study, LPS-induced NET formation in mice led to increased tumor size, which could be reversed by DNase I or a p38 MAPK inhibitor. The study suggests that NET formation promotes tumor growth by stimulating angiogenesis, rather than directly increasing cancer cell proliferation.

It is well known that the acquisition of gain-of-function mutations allow cancer cells to alter their proliferative capacity as well as their ability to migrate and invade tissues. The effect of neutrophil-generated NETs on cancer cells proliferation, invasion and migration has been intensively studied. Studies show that the effect of NETs on proliferation is cell dependent. For example, it has been shown that NETs have no effect on the proliferation of gastric cancer AGS cells (8, 78), whereas they stimulate the proliferation of glioma LN229 cells (79), colon cancer cells MC38 (80) and HT29 (72). In contrast to conflicting reports on the effects of NETs on cell proliferation, the available data are consistent in showing a stimulatory effect of NETs on cell migration and invasion (15, 72, 79–83).

### 6.2 NETosis and metastasis formation

NETS may also promote cancer progression by supporting the mechanism of metastasis. Studies have shown that NETs can promote cancer metastasis in several ways: by promoting epithelial to mesenchymal transition, by stimulating the adhesion of circulating cancer cells to the tissue of the metastatic organ, by awaken dormant cancer cells, but also by shielding cancer cells from immune cells. Epithelial to mesenchymal transition (EMT) is a process where cancer cells lose cell–cell adhesion, enabling them to migrate, invade adjacent tissues, and metastasize. This process is characterized by the downregulation of epithelial markers like E-cadherin, ZO-1, ocludin and the upregulation of mesenchymal markers such as N-cadherin, vimentin and fibronectin (84). Studies have shown that NETs can promote EMT in cancer cells (8, 85). For example, Martins Cardoso et al. found that treating MCF7 cells with NETs led to a fibroblast-like shape, decreased E-cadherin, and increased N-cadherin and fibronectin (9). These changes were linked to higher expression of EMT-related transcription factors ZEB1 and Snail (86). Zhu et al. further demonstrated that NETs promote EMT in AGS gastric cancer cells and that inhibiting NET formation can reverse these EMT marker changes *in vivo*. Their findings suggest that NET formation may enhance EMT and promote metastasis in gastric cancer cells (8). A second way in which NET formation may promote cancer metastasis is by stimulating the adhesion of circulating cancer cells to the tissue of the metastatic organ and the growth of the metastatic tumor. NETs present in metastatic organs such as the liver may promote cancer cells extravasation (81). After intraperitoneal injection of PMA to induce NET formation in BALBc nude mice, followed by intrasplenic injection of PANC1 pancreatic cancer cells, Kajioka and colleagues observed that more pancreatic cells extravasated into the livers of mice with liver NETs compared to vehicle-injected controls. Similar conclusions can be drawn from the work of Tohme and colleagues (80). They used a murine model of liver ischemia-reperfusion surgery, known to induce inflammation and hepatic metastases. They confirmed that this surgery leads to significant NET formation in the liver. Mice with liver ischemia-reperfusion and intrasplenic injection of colon cancer cells developed more liver metastases compared to sham-operated controls. DNase I treatment reduced NETs and metastasis levels, indicating that NET formation enhances micrometastatic foci and supports the growth of preexisting liver tumors. Cools-Lartigue et al. investigated whether NETs capture circulating tumor cells (CTCs) and promote their adhesion to distant organs in the context of severe infection using a cecal ligation and puncture (CLP) model sites (82). They found increased NET deposition in the hepatic and pulmonary microvasculature after CLP, leading to a higher number of metastatic liver nodules compared to controls. NET inhibitors like DNase I reduced liver metastases, and *in vivo* imaging showed cancer cells trapped by NETs in the liver and lungs. Yang et al. explored whether NET DNA acts as a chemotactic factor for cancer cells. They identified CCDC25 (Coiled-coil domain containing 25) on cancer cell membranes as binding to NET DNA, triggering a signaling cascade that promotes metastatic growth (87). CCDC25, a transmembrane protein containing a coiled-coil domain, functions as a receptor that detects extracellular DNA, particularly DNA derived from neutrophil extracellular traps (NETs), which is rich in oxidized DNA fragments. Upon binding of NET-DNA to the extracellular domain of CCDC25, an intracellular signaling cascade is activated, involving integrin-linked kinase (ILK), the adaptor protein β-parvin, and the small GTPase RAC1. Activation of ILK leads to the recruitment of β-parvin, which facilitates signal transmission to RAC1. RAC1, as a key regulator of the actin cytoskeleton, initiates actin filament reorganization, resulting in the formation of cellular protrusions (filopodium-like protrusion), enhanced cell migration, and increased invasive capacity of cancer cells A third possible pathway by which NET formation can promote cancer metastasis was described by Albrengues and colleagues who showed that inflammation-stimulated NET formation can awaken dormant cancer cells (10). Using murine models of cancer cell dormancy, they found that breast cancer cells remained dormant in the lungs for up to 8 months. However, inducing lung inflammation with LPS or tobacco smoke led to cancer cell awakening, increased proliferation, and aggressive lung metastasis. Neutrophil activation and NET formation were involved in this process. DNase I treatment, which inhibits NET formation, prevented the awakening and metastatic growth of these cells. Additionally, NET-associated proteases like NE and MMP9 were found to remodel the extracellular matrix, stimulating cancer cell awakening through integrins α3 and β1 and yes-associated protein 1.

Another possible means by which NET formation may promote cancer growth is by shielding cancer cells from immune cells. A nice illustration of this phenomenon was provided by the study of Tejeira and colleagues (59). In one of the experiments performed, the researchers investigated the effect of NETs on the interaction of small tumor organoids with cytotoxic T CD8+ cells or natural killer cells and found that NETs protected the spheroids from cytotoxicity and increased the number of surviving cancer cells. When NETs were disrupted by the treatment with DNase, T cells and NK cells exerted their cytotoxic effect on tumor cells. Using imaging techniques, the researchers showed that extruded NETs surrounded cancer cells and prevented access to them. Intravital microscopy experiments confirmed that this phenomenon can also occur *in vivo*.

## 7 Endocannabinoid system

Discovered in the late 1980s, the endocannabinoid system (ECS) is crucial for maintaining bodily homeostasis. Initially, it was thought that phytocannabinoids, like THC, acted by altering cell membranes. However, by 1988, radiolabeling techniques revealed high-affinity cannabinoid receptors in rat brain membranes using the radiolabeled cannabinoid CP55940, identifying these binding sites as crucial components of the ECS (88–90). Initially, it was believed that the ECS primarily regulated the nervous system; however, subsequent research has elucidated its role in governing key physiological processes such as anxiety, appetite regulation, the reward system, pain perception, fertility, immune system, and numerous other vital functions (91, 92). The ECS system consists of three main components: endogenous cannabinoids, enzymes involved in their biosynthesis and degradation, and endocannabinoid receptors. ECS receptors are ubiquitously distributed in various anatomical sites, including the central nervous system, pulmonary system, gastrointestinal tract, skeletal system, reproductive organs, and peripheral nervous system. Three different classes of endocannabinoid receptors are recognized within the ECS: CB1 and CB2 receptors, which are G protein-coupled ligand-gated ion channels, and nuclear receptors. CB1 receptors are predominantly located in the central nervous system, while CB2 receptors are expressed primarily in immune cells. Other endocannabinoid receptors include G protein-coupled receptors such as GPR18, GPR55, and GPR119 (93, 94). The endogenous ligands for CB1 and CB2 receptors are 2-arachidonoylglycerol (2-AG) and N-arachidonoylethanolamine (AEA), which act as agonists or antagonists for these receptors. Upon ligand binding, the conformation of the associated G protein is altered, initiating a signaling cascade that triggers specific physiological responses, such as the inhibition of neurotransmitter release (95). The enzyme diacylglycerol lipase (DAGL) catalyzes the synthesis of 2-AG, whereas AEA is synthesized by the hydrolysis of N-acylphosphatidylethanolamine by phospholipase D (NAPE-PLD). The degradation of 2-AG is mediated by monoacylglycerol lipase (MAGL) and the alpha/beta hydrolase domain-containing proteins ABHD6 and ABHD12. AEA hydrolysis is facilitated by fatty acid amide hydrolase (FAAH) (96). Endocannabinoid transport is mediated by several proteins, including fatty acid binding proteins (FABPs) and heat shock proteins (HSP70), which are involved in the transport of AEA. Other endocannabinoid transporters include FAAH-like AEA transporters and possibly AMT transporters (97).

ECS receptors can interact not only with endocannabinoids but also with phytocannabinoids derived from plants and synthetic cannabinoid analogs (96). This interaction enables the modulation of ECS activity with a diverse array of compounds, eliciting specific metabolic effects in target cells. Consequently, this modulation can influence various physiological processes, such as pain management, the control of epileptic seizures, and the treatment of depression. Of therapeutic significance is the expression of ECS receptors in both normal and cancerous cells (98–100). This widespread expression suggests that endocannabinoids could be utilized to enhance anticancer therapies, potentially augmenting the efficacy of conventional treatments. Research indicates that the ECS, by modulating interactions between cancer cells and bone cells, can inhibit metastasis formation. Khunluck et al., 2022 (101) investigated the effects of ACEA and GW405833 (agonists of CB1 and CB2 receptors respectively) on MDA-MB-231 breast cancer cells and osteoblast-like UMR-106 cells. Their findings revealed that the conditioned media from MDA-MB-231 cells decreased the viability of UMR cells, while preincubation of MDA-MB-231 cells with GW405833 mitigated this effect. Furthermore, the coactivation of ECS receptors exhibited cytotoxic effects on MDA-MB-231 cells, inducing apoptosis via the inhibition of the NF-κB signaling pathway through a reactive oxygen species (ROS)-independent mechanism. Research by Laezza et al., 2020 (102) indicates that endocannabinoids can mitigate the invasive phenotype of cancer cells by modulating the epithelial-mesenchymal transition (EMT) mechanism. Their study revealed that treating MDA-MB-231 cells with methyl-F-anandamide significantly reduces the levels of cytoplasmic and nuclear β-catenin, resulting in the inhibition of the transcriptional activity of the β-catenin signaling marker T-cell factor (TCF). Additionally, anandamide treatment elevated E-cadherin levels while reducing the expression of mesenchymal markers such as vimentin and Snail1. It was also noted that anandamide inhibited the EMT transition in MCF-7 cells treated with adriamycin.

Stimulation of ECS receptors by cannabinoids in cancer cells can induce apoptosis or inhibit cellular proliferation. In breast cancer, a heteromeric complex is formed between the CB2 receptor and the epidermal growth factor receptor 2 (HER2) and the expression of this complex correlates with poor disease prognosis. However, this complex also presents a viable therapeutic target. Notably, THC has been observed to disrupt the formation of these heteromers by selectively binding to the CB2 receptor. This binding leads to the inactivation of HER2 and its subsequent degradation via the proteasome E3 ligase c-CBL pathway (103).

In triple-negative breast cancer, cannabinoids interacting with the CB1 and/or CB2 receptors confer a less metastatic phenotype and may inhibit cellular proliferation. Song et al., 2023 (104) observed that overexpression of the CB2 receptor in breast cancer cells, coupled with treatment using a CB2 receptor agonist, inhibits cell proliferation and promotes apoptosis. This effect occurs through inhibition of the PI3K/Akt/mTOR signaling pathway. Additionally, studies have shown that anandamide and the paracannabinoid lysophosphatidylinositol (LPI) exert opposing effects on breast cancer cell proliferation: anandamide inhibits proliferation, while LPI stimulates it. Research by Akimov et al., 2024 (105) indicates that the cytotoxic effect of anandamide (AEA) is mediated through the CB2 receptor, whereas LPI enhances signaling through the GPR18 receptor and mitigates AEA-induced cell death. Some studies also suggest that, via various mechanisms either dependent on or independent of ECS receptors, CBD exerts a direct effect on lung cancer cells (106, 107).

In addition to its direct effects on cancer cells, the endocannabinoid system (ECS) can indirectly regulate carcinogenesis by modulating the tumor microenvironment, particularly the immune system cells (108).

Of particular interest is the influence on neutrophils, which are excessively activated in the tumor microenvironment. These granulocytes significantly contribute to the metastatic process. The impact of the ECS on their effector functions will be discussed in greater detail in the subsequent chapter.

## 8 Phytocannabinoids

**Figure 2:**
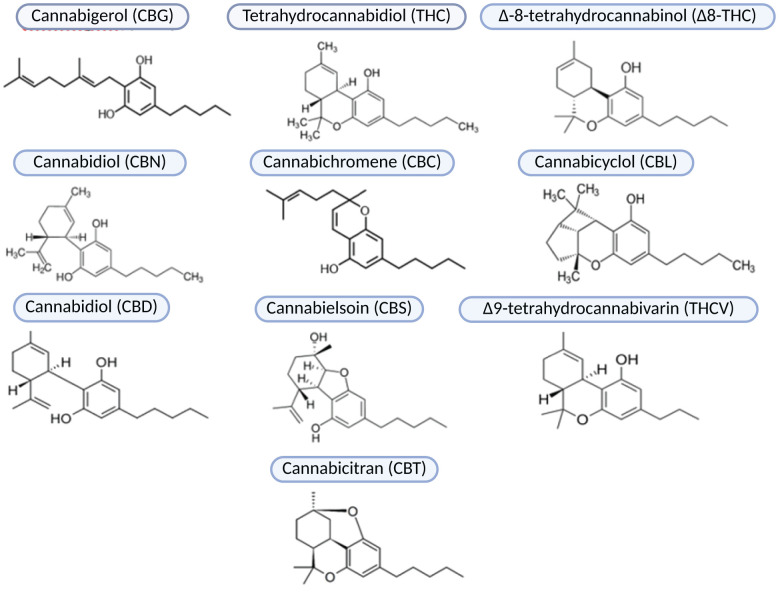
Phytocannabinoids subclasses.

Known for its psychoactive properties, hemp has received considerable attention and controversy in research. However, certain varieties of this plant lack psychoactive effects and possess numerous biologically active compounds with potent antioxidant and anti-inflammatory properties. There is growing interest in using these non-psychoactive cannabis strains as therapeutic agents for a wide range of diseases are steadily increasing. *Cannabis sativa* is the most extensively studied plant species known for its rich reservoir of phytocannabinoids. This plant contains a diverse array of approximately 540 compounds with bioactive properties, including over 100 phytocannabinoids. These phytocannabinoids are classified into ten subclasses that include degradation products, precursors, and intermediates, including CBG (Cannabigerol), THC (Tetrahydrokannabinol), Δ8-THC (Δ-8-tetrahydrocannabinol), CBN (Cannabidiol), CBC (Cannabichromene), CBL (Cannabicyclol), CBD (Cannabidiol), CBE (Cannabielsoin), THCV (Δ9-tetrahydrocannabivarin), and CBT (Cannabicitran) (**Figure 2**).

Phytocannabinoids have a characteristic terpene-phenolic structure. Their biosynthesis within hemp plants starts with precursors containing 21 or 19 carbon atoms, such as cannabigerolic or cannabigeranoic acid. Through a series of enzymatic transformations and decarboxylations, these precursors are converted to the final structures of phytocannabinoids (11). In addition to THC, the second most abundant non-psychoactive phytocannabinoid in *Cannabis sativa* is cannabidiol (CBD) (109, 110). The major therapeutic effects of CBD include analgesic, anxiolytic, antidepressant, anticonvulsant, antioxidant, anti-inflammatory, antibacterial, immunomodulatory, and anticancer activities (99, 111, 112). Phytocannabinoids, including CBD, exhibit both direct and indirect anticancer properties. Directly, these compounds exhibit antiproliferative and pro-apoptotic effects and promoting programmed cell death. In addition, phytocannabinoids inhibit cancer cell migration and angiogenesis, thereby attenuating the metastatic process.

CBD has been shown to inhibit proliferation and induce apoptosis in MDA-MB-231 breast cancer cells both *in vitro* and *in vivo*. These anticancer effects are mediated through the activation of the endocannabinoid CB2 receptor and the vanilloid transient receptor (113). CBD effectively inhibited the growth of xenograft tumors in murine models transplanted with human MDA-MB-231 cells. Additionally, its precursor, cannabidiolic acid (CBDA), inhibits breast cancer cell migration by modulating cyclooxygenase-2 expression and activity (114) and inhibiting protein kinase A (PKA) (115). CBD disrupts the life cycle of cancer cells, leading to apoptosis (116). The anticancer effects of phytocannabinoids are a key focus in both scientific and clinical research (117, 118). Notably, both CBD and THC show significant potential in treating lung cancer by influencing apoptosis, invasion, and adhesion of cancer cells. For example, Ramer et al. (2012) (119) found that CBD inhibits lung cancer cell invasion by inducing tissue inhibitor of metalloproteinases-1 (TIMP-1) through intercellular adhesion molecule-1 (ICAM-1) activation. Haustein et al. (2014) (120) reported that CBD upregulates ICAM-1 expression on lung cancer cells, enhancing their adhesion to lymphokine-activated killer (LAK) cells, leading to a cytotoxic effect. Additionally, Ramer et al. (2013) (121) observed that CBD induces apoptosis in lung cancer cells by upregulating cyclooxygenase-2 (COX-2) and peroxisome proliferator-activated receptor gamma (PPAR-γ), resulting in apoptotic cell death via nuclear translocation of PPAR-γ. CBD has been shown to suppress angiogenesis and diminish the metastatic properties of breast cancer cells via the Src*/VHL/HIF-1alpha* signaling pathway (Sarcoma, Hypoxia-inducible factor 1-alpha, Von Hippel-Lindau Tumor Suppressor) (122). CBD’s potential to inhibit metastasis is particularly significant for breast cancer, known for its tendency to spread to multiple sites. García-Morales et al. (2023) (123) demonstrated that *in vivo* treatment with CBD reversed EMT transition and malignant phenotype acquisition in MCF-7 cells induced by IL-1β. In mouse models, CBD treatment significantly reduced tumor size, with 66% of the animals showing complete tumor regression. Histological and molecular analyses revealed decreased malignancy markers and increased tumor cell apoptosis, highlighting CBD’s therapeutic potential in breast cancer by mitigating metastasis and promoting tumor regression. A similar effect was observed in non-small cell lung cancer (NSCLC), where CBD inhibited proliferation and metastasis of drug-resistant NSCLC via the TRPV2 ion channel receptor. Additionally, CBD promoted lung adenocarcinoma cell apoptosis by modulating the oxidative stress pathway (124).Studies have also documented that cannabidiol (CBD) interacts with immune system cells, which form a substantial component of the tumor microenvironment (125). Phytocannabinoids have been shown to modulate the activity of various immune cells. However, there is a paucity of research investigating the direct effects of phytocannabinoids on immune cells, particularly neutrophils, which are the primary focus of this review.

### 8.1 Phytocannabinoids, NETosis, and tumor metastasis

The presence of endocannabinoid receptors, and in particular CB2R, on neutrophils was first confirmed by Galiègue S at all., 1995 (126). This discovery initiated studies aimed at understanding the function of endocannabinoid receptors in the context of neutrophil functioning. It turned out that particularly the peripheral CB2 receptor (CB2R) can modulate the effector functions of neutrophils like activation, migration, degranulation or superoxide generation (127). Literature indicates that while CB2R expression is relatively low in neutrophils of healthy individuals, it can be upregulated in inflammatory conditions or in response to phytocannabinoid exposure (128–130).

Wójcik et al. investigated the direct effects of phytocannabinoids on neutrophil activation and NETosis in a psoriasis model. Their research indicates that CBD may reduce NETosis markers, including myeloperoxidase (MPO) and NADPH oxidase. CBD also lowered cell-free DNA (cfDNA) levels, correlating with a reduction in NETosis, though it did not completely eliminate the pro-inflammatory phenotype of neutrophils (13). Furthermore, several studies have indirectly assessed the impact of phytocannabinoids on neutrophil effector functions like chemotaxis, degranulation, oxidative burst and the NETosis process by evaluating their influence on classical NETosis markers. In a study by Gómez et al. (2021) (131), the effects of CBD on neutrophil function were examined. The findings revealed that CBD reduces fMLP-induced neutrophil chemotaxis, decreases oxygen consumption and H2O2 production, but promotes the release of singlet oxygen, a reactive oxygen species. CBD modulated neutrophil functionality in a concentration-dependent manner, with excessive concentrations leading to vacuolization of the polymorphonuclear (PMN) cytoplasm, pro-apoptotic nuclear condensation, and reduced cell viability. Tagne et al. (2019) (14) investigated the effects of CBD and a novel CM5 extract from Cannabis sativa on fMLP-activated neutrophils. Both CBD and CM5 significantly reduced neutrophil migration, ROS production, and TNF-α levels. CBD was more effective than CM5 in modulating PMN oxidative metabolism and reducing neutrophil activation. Cannabinoids have been shown to modulate fMLP-stimulated neutrophil recruitment and migration. In an LPS-induced acute pneumonia mouse model, THC significantly inhibited neutrophil migration and reduced neutrophil elastase, TNF-alpha, and IL-6 levels (132). Studies on gastrointestinal epithelium damage caused by HIV/SIV suggest that low concentrations of THC may reduce neutrophil infiltration by modulating MMP25-AS1 and decreasing MMP25 expression (133). In a murine pneumonia model, a high-CBD extract significantly reduced neutrophil migration to the lungs and lowered pro-inflammatory cytokines like IL-1β, MCP-1, IL-6, and TNF-α (134). These findings emphasize CBD’s strong anti-inflammatory and immunomodulatory properties, suggesting its therapeutic potential in inflammatory conditions. In LPS-induced lung inflammation models, both CBD and CBG significantly reduced neutrophil infiltration (135) (136). CBG and its derivatives—HUM-223, HUM-233, and HUM-234—were evaluated for anti-inflammatory and analgesic effects in preclinical murine models. HUM-223 notably reduced the expression of genes like Adams4, neutrophil elastase (Elane), and myeloperoxidase (MPO), highlighting the therapeutic potential of phytocannabinoids in modulating inflammation and preserving tissue integrity. Studies on murine liver damage from excessive alcohol consumption found that CBD attenuates the oxidative burst of neutrophils (128). Further research showed that CBD inhibits PMA-induced neutrophil activation in a concentration-dependent manner, with this effect persisting despite a CB2 receptor antagonist, indicating CB2-independent inhibition. Furthermore Naccache et al. (1982) (137) studied the effects of cannabinoids on rabbit neutrophil degranulation, found that CBD and THC influenced lysosomal enzyme release in a dose-dependent manner. Notably, CBD induced faster degranulation than PMA stimulation. These findings highlight phytocannabinoids’ ability to regulate neutrophil functions, including extracellular trap release. While CBD shows therapeutic promise, current evidence is insufficient to confirm its direct impact on neutrophil activation in the tumor microenvironment, warranting further research.

**Figure 3:**
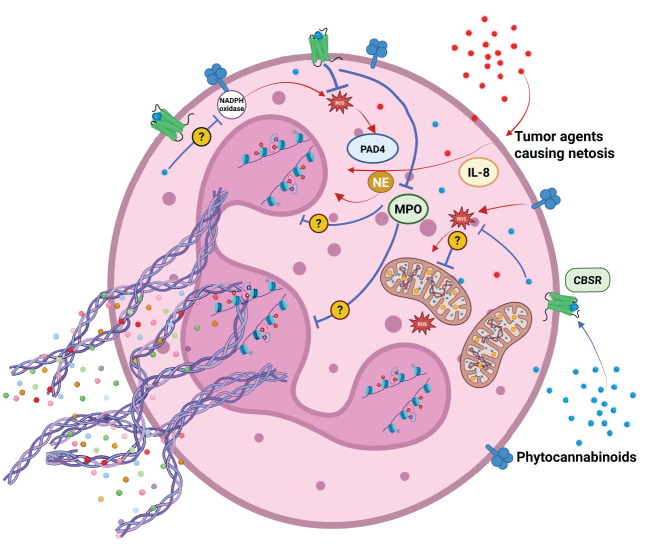
Agents released by cancer cells support neutrophil activation and NETosis. Phytocannabinoids can modulate NETosis induction by several mechanisms. They can: reduce ROS production, oxidative burst, inhibit MPO and NADPH oxidase activity. Their action may or may not be dependent on endocannabinoid system receptors CBSR.

One *in vivo* study suggests that THC may reduce macrophage and neutrophil infiltration in skin cancer (134). Research by Baban et al. (2018) (138) shows that cannabidiol increases regulatory T cells and polarizes neutrophils to the immunosuppressive N2 phenotype in acute kidney inflammation, offering renoprotective benefits. Phytocannabinoids are believed to have therapeutic potential mainly due to their antioxidant and anti-inflammatory properties, though their precise mechanisms remain unclear (109). The studies suggest cannabinoids may modulate NETosis, which is excessive in the tumor microenvironment, potentially limiting metastasis. However, current research is limited, and further in-depth studies are needed to clarify the cellular and molecular mechanisms involved (**Figure 3**).

### 8.2 ECS system, NETosis, and their link to tumor metastasis

Investigations into the specific impact of endocannabinoid system (ECS) modulation on neutrophil function within the tumor microenvironment remain sparse. Due to the ever-increasing problem of increasing mortality due to cancer metastases, it is extremely important to thoroughly verify the influence of the ECS on neutrophil activation in the cancer microenvironment. Numerous studies have explored the ECS’s impact on neutrophil function in chronic inflammatory diseases. We speculate that similar effects may occur in the tumor microenvironment. This section aims to highlight the ECS’s crucial role in regulating neutrophil functions across various disease models, emphasizing its potential significance in cancer research (139).

Both ECS receptors and endocannabinoids themselves participate in modulating neutrophil function.

ECS receptors are crucial in regulating neutrophil migration and chemotaxis. CB1 receptors mediate neutrophil chemotaxis and activation in sterile liver inflammation. CB1 activation promotes neutrophil recruitment, ROS release, and liver inflammation. Blocking CB1 receptors reduces neutrophil infiltration and liver inflammation in CCl4-induced liver injury in mice. The study identifies ROS and the p38 MAPK pathway as key to CB1-induced neutrophil activation. CB1 stimulation increases CitH3 expression and DNA release in neutrophils, effects suppressed by the CB1 antagonist AM281. The research suggests targeting CB1 receptors as a therapeutic approach for liver diseases and possibly reducing neutrophil-driven metastasis in cancer (128). In a murine model of *Pseudomonas aeruginosa*-induced pneumonia, CB2 receptor knockout (CB2KO) mice exhibited heightened inflammatory responses and exacerbated pulmonary injury compared to wild-type (WT) mice. Activation of CB2R with the agonist JWH133 reduced immune cell infiltration, particularly neutrophils, while the CB2R antagonist SR144528 reversed this effect. JWH133-treated mice showed reduced neutrophil activation and lower markers of NET formation, such as citrullinated histones, compared to CB2KO mice. These findings suggest CB2R activation modulates neutrophil activation and NETosis, with potential therapeutic implications for controlling inflammation and lung damage in PA-induced pneumonia. This modulation could also be relevant in tumor microenvironments with chronic inflammation (16). Regulating inflammatory responses and tissue damage can limit cancer cell spread from the tumor stroma, impeding metastasis. Activated neutrophils undergoing NETosis secrete angiogenic factors like VEGF, CXCL8, HGF, and MMP-9, which promote angiogenesis and metastasis (140). Emerging evidence suggests that ECS receptors may modulate angiogenesis. In studies with LPS-activated neutrophils, CB1R and CB2R agonists selectively inhibited VEGF-A release without affecting CXCL8 and HGF. These findings highlight the therapeutic potential of targeting the CB-neutrophil axis in inflammatory conditions, including sepsis, asthma, cardiovascular disorders, and cancers (141).

In addition to the CB1R and CB2R receptors the GPR55 receptor is a key player in modulating ECS functions related to neutrophils. Studies using the GPR55 antagonist CID16020046 and agonists (AM251 and Abn-CBD) in ApoE-/- mice models of atherosclerosis revealed that GPR55 modulation affects neutrophil function depending on atherogenic progression and diet. In HD-fed mice, CID16020046 had little effect on neutrophil activity, but in ND mice, it enhanced neutrophil chemotaxis and induced degranulation, which was counteracted by the GPR55 agonist Abn-CBD. These findings suggest GPR55’s role in neutrophil function is context-dependent, and its effects are independent of CB1 and CB2 receptors due to their low expression in neutrophils (142–146). ECS receptors can significantly inhibit human neutrophil migration, though this effect is not mediated by CB1 receptors. Evidence suggests the involvement of a distinct non-CB1, non-CB2 receptor sensitive to antagonism by specific compounds, potentially different from known cannabinoid receptors. This novel pharmacological target, antagonized by N-arachidoloyl L-serine, may have therapeutic implications for modulating the endocannabinoid system in inflammatory conditions (146).

Endocannabinoids like anandamide (AEA) and 2-arachidonoylglycerol (2-AG) promote neutrophil functions such as chemotaxis and phagocytosis. Chouinard et al. (2013) (147) showed that 2-AG activates human neutrophils via hydrolysis to arachidonic acid (AA), leading to LTB4 biosynthesis and BLT1 receptor activation, resulting in the release of antimicrobial factors like IL-37 and alpha-defensin against pathogens such as S. aureus, E. coli, HSV-1, and RSV. Although 2-AG did not induce neutrophil migration, it mobilized migratory activity via LTB4. Kurihara et al. (2006) found that 2-AG inhibited fMLP and CXCL8-induced neutrophil migration, suggesting 2-AG’s role in modulating neutrophil movement (148).

**Figure 4:**
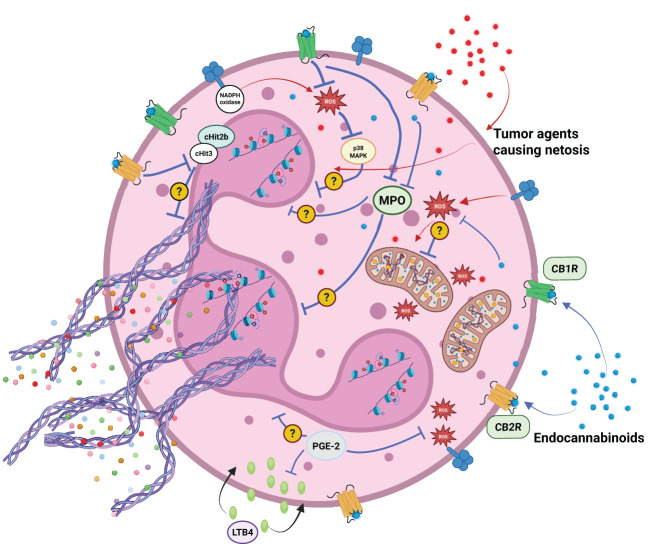
Factors secreted by cancer cells can facilitate neutrophil activation and the formation of neutrophil extracellular traps (NETosis). Elements of the endocannabinoid system (ECS) — including endocannabinoids and their metabolites — may modulate neutrophil activation through ECS receptor-dependent and independent mechanisms. Potential effects include attenuation of reactive oxygen species (ROS) production, suppression of the respiratory burst, inhibition of myeloperoxidase (MPO) activity, inhibition of leukotriene B4 (LTB4) activation and histones citrullination (H3 - citH3 and H2B -citH2B).

In atherosclerosis models, elevated 2-AG levels were found to promote macrophage and neutrophil infiltration into the vascular wall, a process mitigated by CB2 receptor inhibition, underscoring the ECS’s regulatory role in atherogenesis (149). Similarly, in idiopathic enteritis, decreased endocannabinoid levels or loss of CB2 receptor expression correlated with increased neutrophil transmigration, worsening the condition in acute enteritis models (150). Endocannabinoid metabolites can influence neutrophil functions beyond migration. In fibromyalgia (FM), Kaufmann et al. (2008) reported elevated serum anandamide levels, which correlated with enhanced adhesive and phagocytic functions of neutrophils (151). Additionally, the stable anandamide analogue, methanandamide, was found to stimulate the neutrophil respiratory burst via the CB2 receptor, while anandamide itself did not, likely due to its rapid hydrolysis (143). Prostaglandin E2 (PGE2), an endocannabinoid derivative dependent on COX-2, is a known inhibitor of neutrophil effector functions, including leukotriene B4 (LTB4) biosynthesis, ROS production, and neutrophil migration (152, 153). Similarly, PGE2-glyceryl ester (PGE2-G) and prostaglandin D2-glyceryl ester (PGE2-EA) also inhibit LTB4 biosynthesis, superoxide production, antimicrobial peptide release, and neutrophil migration (154). Additionally, othe studies have shown that endogenous cannabinoids like AEA and MethAEA do not affect neutrophil burst responses at physiological levels (143) (**Figure 4**).

## 9 Conclusion

Both phytocannabinoids, especially CBD, and the endocannabinoid system (ECS) show significant therapeutic potential in cancer treatment. Research indicates that these agents affect the proliferation, apoptosis, migration, and invasiveness of cancer cells. In addition, they modulate the tumor microenvironment, particularly the cells of the immune system. Some evidence suggests that these factors regulate the effector functions of neutrophils, which play a critical role in cancer progression and metastasis. However, direct evidence identifying the impact of phytocannabinoids and the ECS on polymorphonuclear neutrophils (PMNs) migrating to the tumor microenvironment remains insufficient.

As cancer metastasis is the leading cause of cancer-related mortality, it is imperative to elucidate the mechanisms underlying neutrophil activation and the subsequent release of neutrophil extracellular traps (NETs), that promote metastasis. Understanding whether phytocannabinoids and the ECS can attenuate NETosis in neutrophils within the tumor microenvironment is of paramount importance. Further investigation into this area is urgently needed to determine the potential of these agents to reduce neutrophils NETosis and thereby inhibit the metastatic process.
