# Correction: Neutrophil extracellular traps and cannabinoids: potential in cancer metastasis

**DOI:** 10.3389/fonc.2025.1663602

**Published:** 2025-08-19

**Authors:** Izabela Krauze, Beata Greb-Markiewicz, Anna Kłopot, Kamila Maciejewska, Michał Bryk, Małgorzata Krzystek-Korpacka

**Affiliations:** ^1^ Department of Biochemistry and Immunochemistry, Wroclaw Medical University, Wroclaw, Poland; ^2^ Department of Biochemistry, Molecular Biology and Biotechnology, Wroclaw University of Science and Technology, Wroclaw, Poland

**Keywords:** phytocannabinoids, endocannabinoid system, neutrophils, NEtosis, cancer metastasis

An incorrect Funding statement was provided. The correct funder is [insert Funder Name]/the correct funding statement reads:

“The author(s) declare that financial support was received for the research and/or publication of this article. This manuscript was published with financial support from Wroclaw Medical University and Wroclaw University of Science and Technology.”

The original version of this article has been updated.

